# Supplementary figures and images for: Transcriptome analysis of gibberellins and abscisic acid during the flooding response in Fokienia hodginsii
Source: PLoS One. 2022 Feb 11;17(2):e0263530. doi: 10.1371/journal.pone.0263530 (PMC8836328; doi:10.1371/journal.pone.0263530)

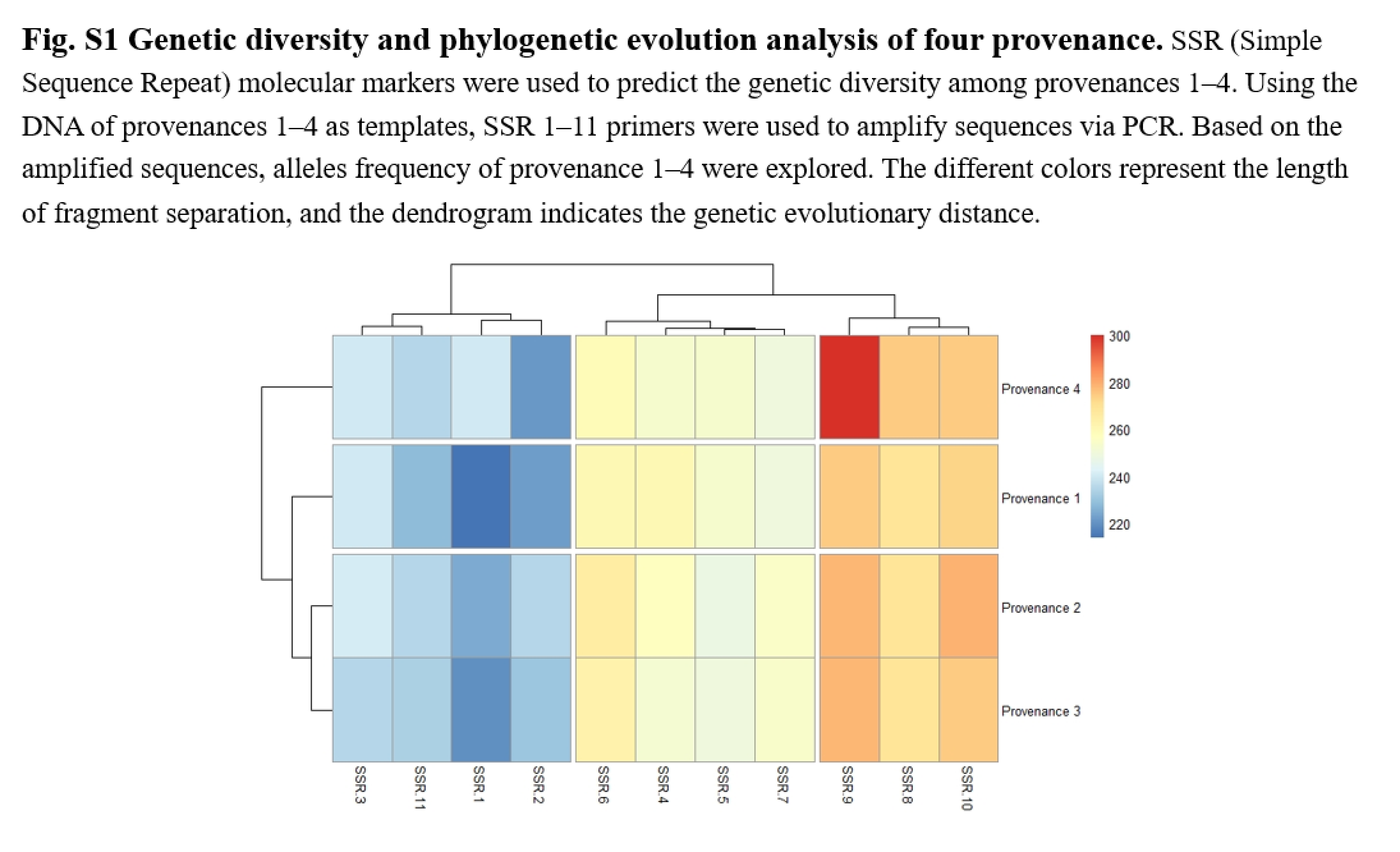

Supplement: S1 Fig — SSR (Simple Sequence Repeat) molecular markers were used to predict the genetic diversity among provenances 1–4. Using the DNA of provenances 1–4 as templates, SSR 1–11 primers were used to amplify sequences via PCR. Based on the amplified sequences, alleles frequency of provenance 1–4 were explored. The different colors represent the length of fragment separation, and the dendrogram indicates the genetic evolutionary distance. (TIF) [file pone.0263530.s001.tif]
