# Supplementary material for: Transcriptome analysis of gibberellins and abscisic acid during the flooding response in Fokienia hodginsii
Source: PLoS One. 2022 Feb 11;17(2):e0263530. doi: 10.1371/journal.pone.0263530 (PMC8836328; doi:10.1371/journal.pone.0263530)
Supplement: S1 Table — (DOCX) [file pone.0263530.s002.docx]

**Tab. S1 List of primers for SSR and RT-qPCR used in this research**

|  | **Name** | | | | **Primers (5’-3’)** |
| --- | --- | --- | --- | --- | --- |
| Primers for SSR | | | FJB5-F  FJB5-R  FJB6-1-F  FJB6-1-R  FJB6-2-F  FJB6-2-R  FJB7-F  FJB7-R  FJB14-F  FJB14-R  FJB23-F  FJB23-R  FJB42-F  FJB42-R  FJB63-F  FJB63-R  FJB73-F  FJB73-R  FJB83-F  FJB83-R  FJB91-F  FJB91-R | ATTCAGCTGGGGACATGCAA  TTCCAGAATCCCAAACGGCC  AAGACTTCCTGGCCTTTGCA  TTAAGCTCTACCGGCTCCCT  AAGACTTCCTGGCCTTTGCA  TTAAGCTCTACCGGCTCCCT  GCATGCTACTCTCCTCGCTT  TGTGCCGTTGAATTTGAGGC  GCAACAAGTGGCACAGTCAG  TGCCTCACCGTGTAGCATTT  TTCTCCACAGGCCACACATG  TCCTGATCTTCATCCGCTGC  TGCGCAAAAGGGTCACAAAG  GGGCCTACTACAACGCTGTT  GGCAGATTATCCAGGTGCGA  CTGAAAGAGGGGGAAGAGCG  ATCCAGAGGCCTTTTTCGCA  CCATTGCATTGCAGGTTGCT  TAGCACAGGAATGGCCATGG  CAGGCCCCCAACAAAGATCT  GTTTCACAGGCGGAAAACCC  ATGAGAACTCCGCTTCGTCC | |
| Primers for qPCR | | ACT7-RT-F  ACT7-RT-R  TRINITY_DN12721_c0_g1-F  TRINITY_DN12721_c0_g1-R  TRINITY_DN142_c0_g2-F  TRINITY_DN142_c0_g2-R  TRINITY_DN1445_c0_g2-F  TRINITY_DN1445_c0_g2-R  TRINITY_DN5304_c1_g1-F  TRINITY_DN5304_c1_g1-R  TRINITY_DN142_c0_g2-F  TRINITY_DN142_c0_g2-R  TRINITY_DN2777_c1_g1-F  TRINITY_DN2777_c1_g1-R  TRINITY_DN4091_c1_g1-F  TRINITY_DN4091_c1_g1-R  TRINITY_DN7657_c0_g1-F  TRINITY_DN7657_c0_g1-R | | | CTGCAGAGCGAGAAATTGTTCG  TTCCCTTGGCTGTTTCAAGC  TTGTGGCGCTCATGAGATTG  ATAATGCAGCGCCGTTTCAC  TTCAAGCAAGAGAGCCCTGTAG  AAATGGCTGCAAACGTGACG  TTGTTTGCAGCCGTGAAAGG  ACATGAACAACGCACCTTCC  GGAATTGAAGGACAACCAAGGC  ACGAGCTGCTTCACCATATG  TTCAAGCAAGAGAGCCCTGTAG  AAATGGCTGCAAACGTGACG  ATTGCCACCGTGACATTTGC  AGGAAAGCCGAAGTGTCAGAG  TGCCAATTTGTGTGCCTCTG  AATGCAGGGCGTTTGATGTG  ACATGGCAAGTGATGAAGGG  TGTTCCTTCGTGCACTTTGC |
