# Supplementary material for: Transcriptome analysis of gibberellins and abscisic acid during the flooding response in Fokienia hodginsii
Source: PLoS One. 2022 Feb 11;17(2):e0263530. doi: 10.1371/journal.pone.0263530 (PMC8836328; doi:10.1371/journal.pone.0263530)
Supplement: S2 Table — (DOCX) [file pone.0263530.s003.docx]

**Tab. S2 Overlapping genes of ABA and GA**

| **Pathway** | **Gene** | **Length (bp)** |
| --- | --- | --- |
| Regulation of salicylic acid mediated  Regulation of defence response  Cellular response to salicylic acid stimulus | TRINITY_DN12721_c0_g1 | 2087 |
|  | TRINITY_DN142_c0_g2 | 1302 |
|  | TRINITY_DN1445_c0_g2 | 2323 |
|  | TRINITY_DN5304_c1_g1 | 529 |
|  | TRINITY_DN142_c0_g2 | 1302 |
|  | TRINITY_DN2777_c1_g1 | 1205 |
|  | TRINITY_DN4091_c1_g1 | 729 |
|  | TRINITY_DN7657_c0_g1 | 740 |
