# Supplementary material for: Transcriptome analysis of gibberellins and abscisic acid during the flooding response in Fokienia hodginsii
Source: PLoS One. 2022 Feb 11;17(2):e0263530. doi: 10.1371/journal.pone.0263530 (PMC8836328; doi:10.1371/journal.pone.0263530)
Supplement: Attachment — Submitted filename: Response to comments.docx [file pone.0263530.s004.docx]

Reviewer #1.

1. Line 33: For “other three provenances” it may be helpful to specify the country of origin within introduction of the species.

Response: Line 27. This is a very good suggestion. We have already added the regions of origin to specify “other three provenances”.

2. Line 66: Missing a statement on the aim of this study at the end of the introduction section. What is novel about this research? Has physiological and molecular characterizations of *F. hodginsii* not been done before?

Response: Line 74. Thank you for reminding. We have added the statement at the end of introduction.

As far as I know, the research on *F. hodginsii* is mainly focused on genetic evolution and metabolites, and there is very little gene function research.

3. Line 78: Is the flooding assay done with some light or complete darkness?

Response: Line91. It was conducted in complete darkness to minimize the o^2-^ contamination and we have added the condition of this treatment.

4. Line 173: I feel the title “Different ABA- and GA-related response genes” is too simple and can be more elaborated, like “Transcriptomic analyses reveal different…”

Response: Line 222. Thanks for this kind advice, we have changed the title “Different ABA- and GA-related response genes” to “Transcriptomic analyses reveal different ABA- and GA-related response genes”.

5. Line 181: Missing hyphen “ABA related genes”

Response: Line 230. We deeply sorry about this mistake, we have added the hyphen.

6. Line 228: Needs space between “1(“

Response: This less relevant paragraph has been removed based on the comments of other reviewers.

7. Line 228: The jump between paragraphs from GA to SUB1 does not connect with the first sentence. It is more clear when the next sentence mentions GA. This may not be necessary, but perhaps there can be a better transition between the paragraphs.

Response: This less relevant paragraph has been removed based on the comments of other reviewers.

8. Line 236: By the end of the SUB1 paragraph, it does not clarify how the SUB1 work relates to the data obtained. Perhaps a sentence can be added linking the connections between the studies, or how the SUB1 data can help support the current data.

Response: This less relevant paragraph has been removed based on the comments of other reviewers.

9. Line 255: Before you mention the measurements of ABA and GA, you can highlight why you performed this work, the purpose, or how the research is novel. This is interpreted but not clearly stated.

Response: Line 49. In the background section, we have added an elaboration on the selection of ABA and GA.

Fig 1A: The black text in the map is hard to read and the font is small in the box.

Response: Corrections have been made.

Fig 1B: Since the squares are most strongly stacked and slightly bigger than the other symbols, perhaps showing a bar or box & whisker graph might make the data points more clear and easier to interpret.

Response: Corrections have been made.

Fig 2: Missing axis title for hours of submergence.

Response: Corrections have been made.

Fig 3A & C: It would help to draw a line under the provenance 1 & 4 samples in the graph.

Response: Corrections have been made.

Fig 3B & D: Missing axis title for hours of submergence.

Response: Corrections have been made.

Table S1: FJB91-R format needs to be centered.

Response: Corrections have been made.

Reviewer #2.

1. In the M&M section says *F. hodginsii* seedlings were grown in hydroponics and subsequently (2 weeks after germination) completely immersed in water. The same hydroponic medium was used? Tap or clean water (potentially leading to the dilution of nutrients and a nutritional imbalance)? Seedlings were transplanted to soil prior to submergence? This needs to be further elaborated. O_2_ levels need to be measured in these conditions.

Response: Line 89. *F. hodginsii* seedlings were cultured used the same hydroponic medium and the water used treated was purified water, these have been added in the M&M section. About O_2,_ Flooding is a complex abiotic stress, anoxia is one of the important stress caused by flooding, it is reasonable to clarify the O_2_ levels in different conditions. But in our case, we want provide a basic understanding of the flooding, the integrated stress, other than part of it. Moreover, O_2_ level was not always mentioned in similar works, partly because quantify the O_2_ levels may miss lead the audience that anoxia can be isolated from the flooding stress.

2. RNAseq data has to be deposited in a publicly available repository (e.g. NCBI SRA or similar) before manuscript is accepted for publication or upon acceptance, not later.

Response: The sequence data reported in this study have been deposited in the Genome Sequence Archive in BIG Data Center, Beijing Institute of Genomics (BIG), Chinese Academy of Sciences, under accession numbers CRA005262, CRA005262 that are publicly accessible at <https://bigd.big.ac.cn/gsa/browse/CRA005262>.

3. Authors recorded phenotypic data from *F. hodginsii* accessions collected from four different locations, but do not provide data that link these phenotypic traits with improved or decreased tolerance to anoxia. Moreover, phenotype responses to hypoxia were not measured or considered.

Response: Thanks for your advices. As we mentioned earlier, anoxia is one of the stress caused by flooding, not all of it. And, it was well studied that controlling of shoot, petiole, or internode elongation were the phenotypes greatly associated with flooding stress. Moreover, it is impossible to collect phenomics data other than height, diameter at breast height, crown diameter, clear height, and branch angle in the field, considering the complex working condition and poor operability. In this work, phenotypic data from the field is only the hint to leading this research. The GA and ABA content in different conditions were the real “phenotypic data” to explain the transcriptome results.

4. Typical responses to hypoxia are missing (ADH, PDC, SUSY upregulation) as well as induction of ERFVII homologs. This needs to be clearly present in the manuscript.

Response: The RNA-seq of *F. hodginsii* was no reference transcriptome, the information of the proteins available is limited, so we do not have the conditions to explore these target proteins, and we can analyze them according to our current results when the reference genome information is released.

5. Statistical analysis is missing in Figures 1, 2 and 3

Response: Thanks, corrections have been made.

6. RNAseq results need to be provided as supplementary data, including annotation and statistical analyses.

Response: Sequencing analysis and annotations have been uploaded to the appendix.

7. Major aspects:

Ethylene has long been acknowledged as the main regulator of plants' responses to reduced O2 conditions, therefore, it cannot be excluded from the discussion. Interactions of ET with GA and ABA pathways in hypoxic conditions have been recently discussed (González-Guzmán et al. 2021).

Response: Line 283. We have added the interactions of ET with GA and ABA pathways in hypoxic conditions in the discussion section.

A proper phenotypic evaluation of accessions in response to hypoxic conditions is required.

Response: Flooding is a complex abiotic stress, anoxia is one of the important stress caused by flooding, it is reasonable to clarify the O_2_ levels in different conditions. But in our case, we want provide a basic understanding of the flooding, the integrated stress, other than part of it. Moreover, O_2_ level was not always mentioned in similar works, partly because quantify the O_2_ levels may miss lead the audience that anoxia can be isolated from the flooding stress.

The proposal that SA signaling is involved is interesting but needs certain experimental backup. What happens with SA levels? What happens with typical SA-linked responses such as PR1 homolog overexpression? Could authors provide a tentative close relative of TRINITY_DN142_c0_g2 and TRINITY_DN7657_c0_g1?

Response: Line 291. Thank you for the affirmation. We have added in the Discussion section about the functions played by SA in abiotic stresses and future research will also be conducted to explain this issue.

L214-217 'suspect' is not scientific. Authors must provide plausible explanations well-grounded on empirical results.

Response: Line 265. Thank you for the advice, we have changed the 'suspect' to 'infer'.

L228-236 SUB1A is a non-canonical ERFVII which is not regulated by the N-end rule pathway and is present in rice. Does F. hodginsii has any potential ortholog? If not, these is out of context here. Other canonical ERFVII are better candidates to link ET and ABA or GA signaling (e.g. Arabidopsis RAP2.3, for instance).

Response: Indeed, as you said, this paragraph was not convincing, so we have removed it.

For the sake of transparency and to allow replicability of results, transcriptional results must be presented in full, including annotation RPKM and statistics (FDR corrected) as supplementary.

Response: Thanks for reminding. Sequencing analysis and annotations have been uploaded to the appendix.

To confirm that low O2 responses were activated under the experimental conditions described, expression of ET/O2 signaling ortholog elements must be presented along with typical anoxia survival responses (expression and/or enzyme activity of ADH, PDC and SUSY). Likewise, signaling pathways for GAs and ABA also need to be presented (expression of ABA receptors, PP2Cs, SnRKs and ABA-responsive genes such as ABI5, DREB2A, RD29, etc...).

Response: Thanks for the suggestion. The transcriptome of this article is a non-reference transcriptome, compared to the reference transcriptome, functional gene analysis is not as detailed. Therefore, our idea is to screen as well as validate the *F. hodginsii* genes with the highest correlation with water flooding, and it is difficult to provide any other valuable information.

8. Minor aspects:
   L190 Where is Table 2, maybe authors refer to supporting Table S2. Please, amend.
   Please, use the same coloring in Figures 3 and 4, as it stands is misleading.

Response: We deeply sorry about the mistake. We have changed “Table 2” to “Table S2”.

Reviewer #3.

1. Components of the PCR reaction volume doesn’t add up to 20. list them all. (Line 89)
   Used GA standard/s (if used) not specified. There are various forms of GA available in the plants system, which one did you measure? (Line 95-102)

Response: Line 101.Thanks for pointing this out. We have listed “7 µL ddH_2_O”.

Line 107. We detected the contents of endogenous ABA and GA_3_, corrections have been made.

2. The gel electrophoresis will not tell you much/precisely the quality of the RNA. Have you run a the samples on the bioanalyzer? or Nano drop absorbance for these purposes? (Line 105)

Response: The gel electrophoresis is only to detect whether RNA is degraded, and we have measured the RNA concentration, absorbance value, 260/230, 260/280 before reverse transcription.

3. what reference genome did you use to design the primers? (Line 109)
   which qRT-PCR instrument did you use? (Line 108-113)

Response: Line 145.primers were designed by using De novoassembly RNA-seq data (Table S1) and qRT-PCR instrument is Applied Biosystems QuantStudio™ 7 Flex.

4. consistency in writing terminologies. For example qRT-PCR (Line 108) vs RT-qPCR (supporting information line 9. Look into the rest of the manuscript for consistency

Response: Thanks for pointing out, Line145,240,303,325 - we have standardized to “RT-qPCR".

5. How did you calculate the relative GA and ABA amounts in Fig. 2 and 3 (Line 14-19; 22-28)? it should be elaborated in the figure legends and method section.

I recommend to include the hormone analysis data, at least in the supporting information section of the manuscript.

Response: Line 109. In the Materials and Methods section, we have added detailed steps and methods for the determination.

6. Can the authors reanalyse the transcriptomic data considering the quality check used at the begining of the analysis were Q10. It should be at least Q30 with inferred base call accuracy of 99.99% rather than 90% in Q10 (Line 124).

Response: Line 162. Thanks for pointing out, we checked the sequencing results again, sequence splicing assembly is based on Q30 standard to ensure the accuracy of the results.

7. How do you explain that you have supplemented the plants to be analyzed for RNAseq with GA3 but quantification from the other set of experiment stated only endogeneous GA. Can you reanalyze the hormone data by including other GA forms rather than stating indogenoeus GA, considering their que-specific responses?

Response: Thanks for pointing this out. Actually, we only quantified GA_3_ by HPLC-MS since GA_3_ was proved to be the most sensitive form of GA to flooding. Relative changes have been made to clarify that only GA_3_ was quantified in this study
