# Supplementary material for: Transcriptome analysis of gibberellins and abscisic acid during the flooding response in Fokienia hodginsii
Source: PLoS One. 2022 Feb 11;17(2):e0263530. doi: 10.1371/journal.pone.0263530 (PMC8836328; doi:10.1371/journal.pone.0263530)
Supplement: Attachment — Submitted filename: renamed_c3051.docx [file pone.0263530.s005.docx]

General considerations on the manuscript “Transcriptome analysis of gibberellins and abscisic acid during the flooding response in Fokienia hodginsii”

1. At no point in the text is cited the country where the provinces are located. It is implied but not explicit. Furthermore, the denominations of the provinces are ambiguous and change during the text, making the understanding of the reader from another country confusing.

2. In the introduction, in the paragraph beginning on line 71, the flooding conditions that may have triggered the plants' adaptation to excess water are mentioned. It would be interesting to inform the time needed for the studied species to complete a reproductive cycle, since the epigenetic changes triggered by stress might not have established themselves as marks transmitted between generations in a short period of time.

3. In lines 85-87 it is not clear whether the plants were grown under hydroponic conditions or in soil.

4. In line 91, it would be pertinent to add the justification for the growth to have occurred in the dark. Do the authors believe that the gene expression observed in the dark condition reflects the transcript pattern that would be expected for these plants under natural conditions? Furthermore, there is no information on acclimation to the dark condition or the growing temperature.

5. In line 100 there is no information on where the sequences of primers used in the experiment are found.

6. In line 122 there is not enough information about the analysis conditions in UHPLC.

7. In the “Simple Sequence Repeat (SSR) assay” section, there is no information on the methodology for extracting genetic material. How many grams of material were used? Did each sample come from different plants or from a pool of individuals? Couldn't the genetic variability of the species mask the results if different individuals make up the same sample?

8. In the section “Total RNA extraction, cDNA reverse transcription, and quantitative real-time (qRT) PCR” there is no information on the methodology used to extract the genetic material from the sample. How many grams were used in the extraction and which plant organs were collected for this trial? Did each individual compose a sample or was a pool of individuals performed during the extraction? Given the genetic variability of the species, would the pool represent a uniform condition of transcripts during the analyses?

9. In the section “RNA-Seq analysis and bioinformatics” an experiment is mentioned where the seedlings were treated with 1 µM GA3 and 20 µM ABA, but this methodology was not described in the text.

10. In line 159 the conditions for RNA sequencing were not described.

11. The “INITY_DN7657_c0_g1” gene is constantly written without “TR” (lines 36, 248, 250, 308 and 313 for example), and it was not clear if it was a typo or if the name really is “inity, since in the response to Reviewer #2 and in the caption of Figure 4 it was written as “TRINITY_DN7657_c0_g1”.

12. On line 291, correct O2 to O2

13. Between lines 289-296 the role of ethylene in flooding conditions was discussed. Did the authors analyze ethylene-responsive genes in transcriptomic analysis? The same can be pointed out in the discussion of salicylic acid (lines 297-305).

14. In the conclusion, it would be interesting to include the relevance of the results obtained in this work, as well as the future perspective for new experiments.

15. With regard to the images, in Figure 1B the data caption in the graph is in inverse order to the bars in the graphs (in the bars Province 4 appears first, although it is the last in the image caption).

16. It would be interesting to include the title of each graphic in the images (and not just in the caption).

17. Figures 4A and 4B are very small and difficult to read.
