# Supplementary material for: Transcriptome analysis of gibberellins and abscisic acid during the flooding response in Fokienia hodginsii
Source: PLoS One. 2022 Feb 11;17(2):e0263530. doi: 10.1371/journal.pone.0263530 (PMC8836328; doi:10.1371/journal.pone.0263530)
Supplement: Attachment — Submitted filename: Response to comments.docx [file pone.0263530.s006.docx]

Reviewer #4.

1. At no point in the text is cited the country where the provinces are located. It is implied but not explicit. Furthermore, the denominations of the provinces are ambiguous and change during the text, making the understanding of the reader from another country confusing.

Response: Line79. Thank you for reminding. The original country “China” has been added for *F. hodginsii.*

2. In the introduction, in the paragraph beginning on line 71, the flooding conditions that may have triggered the plants' adaptation to excess water are mentioned. It would be interesting to inform the time needed for the studied species to complete a reproductive cycle, since the epigenetic changes triggered by stress might not have established themselves as marks transmitted between generations in a short period of time.

Response: Line 67. “It takes about 5-7 years from germination to flowering, with flowering in March-April and fruiting in October-November” has been added*.* The time of the 1956 flooding was well documented, perhaps it happened before that too, but there is no record of it, so we have no way of knowing. And later seed gardens may have accelerated this genetic effect.

3. In lines 85-87 it is not clear whether the plants were grown under hydroponic conditions or in soil.

Response: Line87. “under hydroponics” has been added.

4. In line 91, it would be pertinent to add the justification for the growth to have occurred in the dark. Do the authors believe that the gene expression observed in the dark condition reflects the transcript pattern that would be expected for these plants under natural conditions? Furthermore, there is no information on acclimation to the dark condition or the growing temperature.

Response: Line 92. “all treatments were performed under complete dark conditions that included 22℃ and 85% relative humidity.” has been added. It was conducted in complete darkness to minimize the O^2-^ contamination. Dark conditions were operated in a darkened room which included 22℃ and 85% relative humidity.

5. In line 100 there is no information on where the sequences of primers used in the experiment are found.

Response: Line99. Thank you for reminding. “Eleven pairs of SSR primers were used to analyse [20] (Table S1)” has been added.

6. In line 122 there is not enough information about the analysis conditions in UHPLC.

Response: Line 129. We have refined the analysis conditions of UHPLC-MRM-MS and “The MRM parameters for each of the targeted analytes were optimized using flow injection analysis, by injecting the standard solutions of the individual analytes, into the ESI source of the mass spectrometer. Several most sensitive transitions were used in the MRM scan mode to optimize the collision energy for each Q1/Q3 pair. Among the optimized MRM transitions per analyte, the Q1/Q3 pairs that showed the highest sensitivity and selectivity were selected as ‘quantifier’ for quantitative monitoring. The additional transitions acted as ‘qualifier’ for the purpose of verifying the identity of the target analytes. Skyline Software were employed for MRM data acquisition and processing.” has been added.

7. In the “Simple Sequence Repeat (SSR) assay” section, there is no information on the methodology for extracting genetic material. How many grams of material were used? Did each sample come from different plants or from a pool of individuals? Couldn't the genetic variability of the species mask the results if different individuals make up the same sample?

Response: Line 96. Approximately 100mg leaf samples were used to extract DNA. They were pool of individuals from same.

8. In the section “Total RNA extraction, cDNA reverse transcription, and quantitative real-time (qRT) PCR” there is no information on the methodology used to extract the genetic material from the sample. How many grams were used in the extraction and which plant organs were collected for this trial? Did each individual compose a sample or was a pool of individuals performed during the extraction? Given the genetic variability of the species, would the pool represent a uniform condition of transcripts during the analyses?

Response: Line151. We have added the detail “500 mg leaves”. The samples were pool of individuals from same.

9. In the section “RNA-Seq analysis and bioinformatics” an experiment is mentioned where the seedlings were treated with 1 µM GA3 and 20 µM ABA, but this methodology was not described in the text.

Response: Line 166. “The seedling were grown under hydroponics, the 1 µM GA_3_ and 20 µM ABA treatments through adding the appropriate amount of powder to the culture solution to achieve the required concentration.” has been added.

10. In line 159 the conditions for RNA sequencing were not described.

Response: Line 172. We have added the conditions for RNA-seq and “RNA-Seq was performed using the Illumina NovaSeq 6000 with 6G of data and Poly (A) RNA from 1 mg total RNA or purified mRNA and purified m6A-containing fragments were used to generate the cDNA libraries, respectively, according to TruSeq RNA Sample Prep Kit protocol. The sample library type was a eukaryotic unstranded specific transcriptome library.” has been added.

11. The “INITY_DN7657_c0_g1” gene is constantly written without “TR” (lines 36, 248, 250, 308 and 313 for example), and it was not clear if it was a typo or if the name really is “inity, since in the response to Reviewer #2 and in the caption of Figure 4 it was written as “TRINITY_DN7657_c0_g1”.

Response: Lines 36, 265, 267, 335, 330 and 349.Thank you for reminding. Corrections have been made.

12. On line 291, correct O2 to O2

Response: Line 308. Thank you for reminding. The “O^2^” has corrected to “O_2_”.

13. Between lines 289-296 the role of ethylene in flooding conditions was discussed. Did the authors analyze ethylene-responsive genes in transcriptomic analysis? The same can be pointed out in the discussion of salicylic acid (lines 297-305).

Response: These two relevant changes were not found in the available data. Due to genomic information is not available, there is no way to answer the above question further.

14. In the conclusion, it would be interesting to include the relevance of the results obtained in this work, as well as the future perspective for new experiments.

Response: Line 351. We describe the relevance of the results and the outlook and “thus may be the reasons for phenotypic variation of *F. hodginsii*. The analysis of phenotypic differences in *F. hodginsii* revealed the intrinsic molecular regulatory mechanisms and provided some reference value for resistance breeding and adaptive evolution.” has been added.

15. With regard to the images, in Figure 1B the data caption in the graph is in inverse order to the bars in the graphs (in the bars Province 4 appears first, although it is the last in the image caption).

Response: Thank you for reminding. We have corrected the figure.

16. It would be interesting to include the title of each graphic in the images (and not just in the caption).

Response: Thank you for your advice. We tried to take your suggestion, but some of the images have more characters and look a bit crowded with the captions.

17. Figures 4A and 4B are very small and difficult to read.

Response: Sorry about this. We have changed the size and sharpness of the images.
